# A Rare Cause of Dizziness and Gait Ataxia: CLIPPERS Syndrome

**DOI:** 10.5334/jbr-btr.997

**Published:** 2016-02-04

**Authors:** Wim Maenhoudt, K. Ramboer, V. Maqueda

**Affiliations:** AZ sint lucas, BE; Department of neurology, AZ Sint Lucas Brugge, BE

**Keywords:** brain, pons, white matter, clippers, steroids

## Abstract

In this paper we report the case of a 52-year-old woman with multiple contrast enhancing lesions associated with a chronic lymphocytic inflammation of the infratentorial structures. We discuss the symptoms, imaging and treatment of this rare disorder, in which magnetic resonance imaging (MRI) has a crucial role in the diagnosis. Early recognition on MRI and radiological follow-up are also important to optimize the treatment.

## Case report

**Figure 1:**
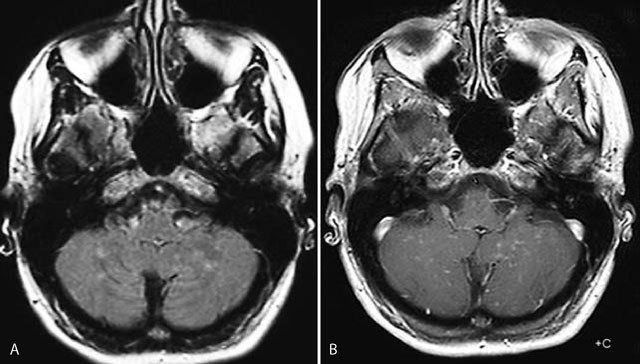
Axial FLAIR image (A): multiple punctate and linear FLAIR hyperintense blurry lesions without mass effect (A) predominatly located in the cerebellar vermis and hemispheres, which become more apperent on contrast-enhanced T1-weighted image (B).

**Figure 2:**
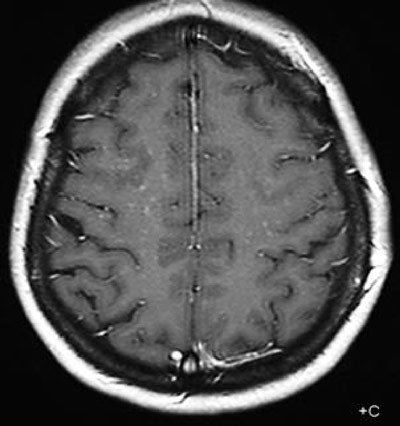
Axial T1 with contrast showing multiple discrete curvilinear lesions in the cerebral white matter of the superior frontal and prerolandic gyrus in both hemispheres.

**Figure 3:**
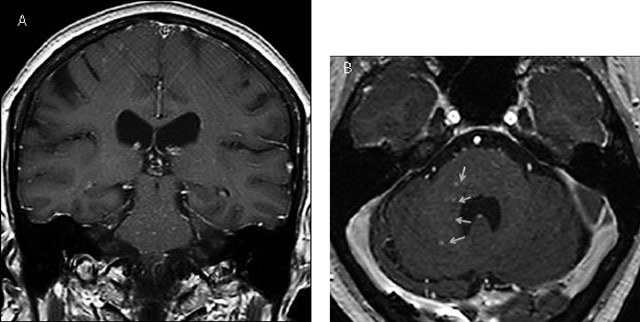
Coronal (A) and axial (B) contrast-enhanced T1 MRI images after 1.5 years showing new punctate and curvilinear lesions predomanintly in the pons and the right middle cerebellar peduncle (yellow arrows).

A 52-year-old patient consults the neurologist with complaints of sustained dizziness, vertigo and unstable gait for more than a month. She has no relevant medical or family history. Clinical neurological examination is normal except for an unstable broad based gait. On electroencephalogram and standard blood examination there were no significant abnormalities. A brain magnetic resonance imaging (MRI) showed multiple areas of minimal fluid attenuation inversion recovery (FLAIR), hyperintense signal and with punctate as well as curvilinear contrast enhancement (Figure 1). These lesions were predominant in the cerebellar vermis and hemispheres. In the prerolandic white matter of both cerebral hemispheres there are similar, yet less pronounced lesions compared to the infratentorial ones (Figure 2). When compared to an older MR preformed nine years ago, these lesions were completely new so a congenital vascular malformation could be excluded. Additionally, more extensive blood tests (paraneoplastic antibodies, rheumatic factors, serologic test for Borrelia, Syphilis and HIV) were ordered as well as an electromyography which all turned out to be negative. Lumbar puncture showed mild lymphocytosis and mildly elevated protein. Cultures of CSF, blood and urine were all negative. A CAT scan of the thorax and abdomen did not show any underlying malignancy. MR of the cervical spine and PET scan were also normal. To exclude the possibility of an intravascular lymphoma, an open biopsy was performed with resection of the most superficial lesion in the cerebellar hemisphere. Biopsy results showed a combined polyclonal B- and T-cell lymphocytes infiltration of the white matter with a largely perivascular distribution which excluded the possibility of a lymphoma. The diagnosis of “Chronic lymphocytyc inflammation with pontine perivascular enhancement responsive to steroids syndrome” (CLIPPERS) was suggested and the patient underwent a treatment with steroids starting with Solumedrol (1g) during five days followed by several months of Prednisone in maintenance dose combined with methotrexate. After three months there was a clinical response to glucocorticosteroids as well as radiological improvement on control MRI. This parallel clinical and radiological evolution, depending on the corticotherapy, confirmed the diagnosis of CLIPPERS. Almost one and a half years later, our patient reported a new episode of pronounced dizziness combined with horizontal nystagmus. A control MRI at that time showed small foci of contrast enhancement in the pons and the cerebral peduncles (Figure 3). The patient was treated with five days of Solumedrol IV (1000mg) followed by a higher maintenance dose. In the following months there was again a good clinical and radiological evolution.

## Discussion

Chronic lymphocytic inflammation with pontine perivascular enhancement responsive to steroids (CLIPPERS syndrome) is a chronic inflammatory disorder of the central nervous system of unknown etiology which was first described in 2010 by Pittock et al. as distinct form of brainstem encephalitis with a predilection for the hindbrain [1]. This condition features brainstem and cerebellar related symptoms combined with a characteristic pattern of gadolinium enhancement on MRI.

Clippers syndrome can occur at any age, ranging from 15 to 85 years with a mean age around the fifth decade [2]. It affects both genders with possibly a minor male predominance. This condition is accompanied by a wide range of symptoms although almost every known patient reports complaints of unstable gait, dysarthria and diplopia in a subacute manner evolving over several weeks. Without treatment the natural course of the disease seems to be relapsing-remitting [2]. Other possible symptoms include dizziness, nausea, tinnitus, tremor, nystagmus, paraparesis, sensory loss and spasticity. Systemic symptoms (fever, weight loss) and symptoms related to connective tissue diseases or rheumaticatic disorders (e.g. arthritis and uveitis) are generally not a feature of this condition and the presence of these symptoms should lead to increased alertness for other causes. Recently the presence of cognitive impairment has been described as a possible long term finding related to CLIPPERS [3].

A crucial role in the diagnosis of CLIPPERS syndrome is preserved for MRI imaging of the brain and spinal cord because it shows a characteristic pattern of punctate and curvilinear enhancement predominantly but not exclusively at the pons and brachium pontis possibly extending in the medulla and midbrain with or without spread in the cerebellar white matter in variable degrees. These individual lesions are very small but may coalesce to form larger lesions [4]. Additionally, lesions may also occur in the cervicothoracal spine cord and supratentorial structures such as the cerebral white matter and the deep cerebral nuclei. On FLAIR images these lesions appear blurry and become more apparent after using contrast. The small hyperintense areas on FLAIR and T2 images do not extend beyond the boundaries of the contrast enhancement which means that the lesions do not cause vasogenic oedema [3]. Normally there is no mass effect but a mild form of swelling of the middle cerebellar peduncle and the pons during relapse has been described [2]. Furthermore the size as well as the number and enhancement of lesions typically decrease with the distance from the pons. Diffusion weighted imaging shows no areas of restricted diffusion and cerebral angiography is normal. PET-CT of the brain normally shows no change or minor hypermetabolism of contrast enhancing areas, considerably less than seen in lymphoma [3]. Although there are only few long term results, patients with CLIPPERS seems to develop significant cerebellar atrophy on follow up MRI even if there is improvement of the inflammatory process and resolution of the contrast enhancement [3].

Next to MR imaging, CAT scan of abdomen and chest, PET-scan, extensive laboratory testing and CSF analysis are necessary for diagnosis, mainly to exclude the possibility of other alternative causes because the list of differential diagnosis is long; especially primary CNS angiitis or lymphoma must be excluded. Other rare conditions which could present themselves in a similar way include neurosarcoidosis, paraneoplastic disease, infectious process (tuberculosis, neurosyphilis, Whipple’s disease, and parasitic infection), glioma, Behcet, Bickerstaff brainstem encephalitis and demyelinating diseases. Some patients initially diagnosed with CLIPPERS syndrome because of the typical clinical and radiological pattern later turned out to be an atypical manifestation of a primary central nervous system lymphoma [2,3].

CSF analysis usually reveals an inconsistent pattern including mild pleocytosis, mild protein elevation and/or oligoclonal bands which, when serially assessed was often observed as a transient phenomenon [2]. If other alternative pathologies cannot be excluded or MR doesn’t show the characteristic pattern, an additional brain biopsy should be performed. The results usually show a perivascular T-cell infiltration, with a predominance of CD4 cells, in the white matter. Although the underlying pathogenesis of this condition is poorly understood and the neuropathological findings are far from specific, these pathology results suggest an inflammatory disorder with a vascular or perivascular tropism predominantly located in the pons and the peripontine region. Some authors suggest that this immune reaction is possibly related to a primary venous inflammatory disorder considering the anatomical arrangement of small intra-axial veins in the brainstem [3]. Nevertheless this theory is based on assumptions and further studies are necessary for better understanding the correlation between the immunological, clinical and radiologic features.

Certainly one of the most important criteria for the diagnosis of this disorder is the clinical as well as radiologic responsiveness to glucocorticosteroid (GCS) based immunosuppression [1,2,3]. Typically the enhancement of the lesions decreases as a result of the therapy. Withdrawal of corticosteroid treatment leads to an exacerbation of symptoms in almost all patients, so maintenance therapy with chronic glucocorticosteroids is required and is the current standard therapy for this condition. The actual treatment of choice is a short course of high-dose IV methylprednisolone followed by oral GCS in combination with an GCS-sparing immunosuppressant. The GCS-sparing agents are used to reduce the daily GCS dose and the chance of GCS side effects. If there is no adequate clinical and radiological response to glucocorticosteroid therapy within the first months, the diagnosis of CLIPPERS should be questioned [2]. Since CLIPPERS has only recently been described as a new disorder, it’s unclear how long maintenance therapy should be continued. Because it has a similar pathologic characteristics as vasculitides, is seems favorable to continue the therapy for at least 2–5 years [4].

## Conclusion

Clippers syndrome is a rare disorder and has only recently been described as a new disease entity in 2010 [1]. Current literature only reports a few cases which raise the question of whether this really is a truly new disease or if it has simply been misdiagnosed for several years as an atypical presentation of known clinically and radiologically similar conditions such as MS, neurosarcoidosis, CNS lymphoma or CNS vasculitis. The disease seems to be centered at the pontine region and the brachium pontis with variable involvement of the adjacent structures. The patients diagnosed with CLIPPERS typically present themselves with a characteristic pattern of brainstem and cerebellar related symptoms. Diagnosis is based on a combination of clinical, radiological and laboratory investigations and, if other conditions such as CNS lymphoma remain a possibility, additional brain biopsy. Another important criterion is the clinical and radiological response to glucocorticosteroids. Therefore glucocorticosteroids in combination with a GCS-sparing agent is the standard therapy. A maintenance therapy combined with a GCS-sparing agent is required to prevent a recurrence of symptoms.

## Competing Interests

The authors declare that they have no competing interests.
